# Filtering of Data-Driven Gene Regulatory Networks Using *Drosophila melanogaster* as a Case Study

**DOI:** 10.3389/fgene.2021.649764

**Published:** 2021-07-28

**Authors:** Yesid Cuesta-Astroz, Guilherme Gischkow Rucatti, Leandro Murgas, Carol D. SanMartín, Mario Sanhueza, Alberto J. M. Martin

**Affiliations:** ^1^Colombian Institute of Tropical Medicine, CES University, Medellin, Colombia; ^2^Centro de Biología Integrativa, Facultad de Ciencias, Universidad Mayor, Santiago, Chile; ^3^Laboratorio de Biologia de Redes, Centro de Genómica y Bioinformática, Facultad de Ciencias, Universidad Mayor, Santiago, Chile; ^4^Programa de Doctorado en Genómica Integrativa, Vicerrectoría de Investigación, Universidad Mayor, Santiago, Chile; ^5^Departamento de Neurología y Neurocirugía, Hospital Clínico Universidad de Chile, Santiago, Chile; ^6^Centro de Investigacíon Clínica Avanzada (CICA), Hospital Clínico Universidad de Chile, Santiago, Chile; ^7^Escuela de Biotecnología, Facultad de Ciencias, Universidad Mayor, Santiago, Chile

**Keywords:** gene regulatory network, transcriptional regulation, transcription factor targets, *Drosophila melanogaster*, *HR96*

## Abstract

Gene Regulatory Networks (GRNs) allow the study of regulation of gene expression of whole genomes. Among the most relevant advantages of using networks to depict this key process, there is the visual representation of large amounts of information and the application of graph theory to generate new knowledge. Nonetheless, despite the many uses of GRNs, it is still difficult and expensive to assign Transcription Factors (TFs) to the regulation of specific genes. ChIP-Seq allows the determination of TF Binding Sites (TFBSs) over whole genomes, but it is still an expensive technique that can only be applied one TF at a time and requires replicates to reduce its noise. Once TFBSs are determined, the assignment of each TF and its binding sites to the regulation of specific genes is not trivial, and it is often performed by carrying out site-specific experiments that are unfeasible to perform in all possible binding sites. Here, we addressed these relevant issues with a two-step methodology using *Drosophila melanogaster* as a case study. First, our protocol starts by gathering all transcription factor binding sites (TFBSs) determined with ChIP-Seq experiments available at ENCODE and FlyBase. Then each TFBS is used to assign TFs to the regulation of likely target genes based on the TFBS proximity to the transcription start site of all genes. In the final step, to try to select the most likely regulatory TF from those previously assigned to each gene, we employ GENIE3, a random forest-based method, and more than 9,000 RNA-seq experiments from *D. melanogaster*. Following, we employed known TF protein-protein interactions to estimate the feasibility of regulatory events in our filtered networks. Finally, we show how known interactions between co-regulatory TFs of each gene increase after the second step of our approach, and thus, the consistency of the TF-gene assignment. Also, we employed our methodology to create a network centered on the *Drosophila melanogaster* gene *Hr96* to demonstrate the role of this transcription factor on mitochondrial gene regulation.

## 1. Introduction

The control of gene expression is one of the key processes that allow living organisms to adapt to their environment. Different regulatory mechanisms determine which gene is expressed and what amount of the product encoded is generated. Among regulatory mechanisms, Transcription Factors (TFs) are deemed to be the most relevant players in the control of transcription, but there are other types of regulation that include ncRNAs and other proteins (Ramírez-Clavijo and Montoya-Ortíz, 2013). TFs bind to specific regions in the DNA to attract or repel RNA polymerase and other components of the transcriptional machinery to modulate the expression of certain genes. When studying the regulation in whole genomes, gene regulation is often represented as a network where nodes represent genes. In this type of network called Gene Regulatory Network (GRN), connections between genes indicate that the product of a gene regulates the expression of another gene, and thus, their direction is important.

Despite the relevance of the processes represented in a GRN, the majority of the different regulators for each gene still remain unknown. For example, in the human GRN there are about 5,400 TF-gene connections of high confidence (Garcia-Alonso et al., 2019), thus, considering there are over 1,600 TFs in this species (Lambert et al., 2018), we still need to verify a large proportion of likely regulators for most of the genes. This lack of knowledge is even worse for other species to a varying degree, including most common model organisms such as *Mus musculus* (Holland et al., 2020), *Caenorhabditis elegans* (Harris et al., 2020), *Drosophila melanogaster* (Thurmond et al., 2019), and even *Escherichia coli* (Santos-Zavaleta et al., 2019). Recent efforts aim to close this gap of knowledge of how genes are regulated. For example, the ENCODE project (Abascal et al., 2020) focuses on the discovery and annotation of cis regulatory elements in human and mouse genomes based on experimental evidence such as TF binding sites. CIS-BP, a database of TF Binding Motifs (TFBMs), employs evolutionary information to infer binding motifs (Weirauch et al., 2014). Another approach to determine TFBMs relies on the detection of motifs from experimentally determined TF Binding Sites (TFBSs) such as those reported by the ENCODE project (Matys et al., 2003; Forrest et al., 2014; Khan et al., 2018; Kulakovskiy et al., 2018). Importantly, even if it is possible to determine where a TFs binds on the DNA by determining occurrences of these motifs (Jayaram et al., 2016), the majority of motifs are not functional (Dror et al., 2015). Even more, the identification of an actual TFBS does not imply knowing which gene or genes are regulated by the binding of the TF to it.

There are several approaches to assign TFs to the regulation of specific genes based on occurrences of TFBMs or experimentally determined TFBMs. Experimental methods to identify TFBSs on DNA are diverse. Non high-throughput methods were initially implemented like DNA footprinting or electrophoretic mobility shift assays (Galas and Schmitz, 1978; Garner and Revzin, 1981; O'Neill and Turner, 1996), these data being a valuable source of several gene regulation databases. According to the genomics advance and DNA sequencing technologies, high-throughput methods were necessary for discovering TFBSs such as Protein binding microarrays, ChIP-chip or ChIP-Seq experiments (Ren et al., 2000; Berger and Bulyk, 2006; Johnson et al., 2007). These methodologies produce large volumes of raw sequence data and different computational strategies need to be implemented for preprocessing and filtering data to find DNA motifs. On the other hand, site-directed mutagenesis (O'Neill et al., 1998) is based on the introduction of modifications in the nucleotide bases that are recognized by the TF residues, restriction enzymes must recognize target sequences with precision to interfere with DNA binding. Nonetheless, once a TFBS is discovered, it still remains to assign its binding to this site to the regulation of a given gene. To do so, one of the techniques is to select targets for a TF if it binds in the respective regulatory region of a gene, e.g., its promoter. Another common way to determine which TFs regulates certain genes is to determine whether their binding motifs or experimentally determined binding sites are near the gene or within a certain distance from the transcription start site (Blatti et al., 2015; Liu et al., 2015; Garcia-Alonso et al., 2019; Qin et al., 2020; Murgas et al., 2021).

There is a fourth approach that aims to assign TFs to genes by identifying regulatory relationships from transcriptional profiles using computational approaches such as GENIE3 (Huynh-Thu et al., 2010) and ARACNE (Margolin et al., 2006). Both tools rely on a relatively large number of transcriptomic experiments, benefiting from the presence of various experimental conditions, and arguable reliability (Marbach et al., 2012; Mochida et al., 2018). While most of these approaches are validated using knowledge driven GRNs such as RegNetwork (Liu et al., 2015), some of the most recent ones employ ChIP-Seq determined TFBSs to estimate their performance (Janky et al., 2014; Desai et al., 2017). Other approaches perform noise reduction in GRNs not only with experimentally determined TFBSs, but also applying GWAS SNPs which are known to alter TF-binding affinities (Chen et al., 2020). Pioneering work in this area related TFBSs to the logfold changes observed in microarray experiments (Bussemaker et al., 2001) or TFs instead of their binding sites once TFBSs were used to assign TF to genes (Gao et al., 2004).

Nowadays, the number of experimentally determined TFBSs keeps steadily growing. This growth is specially relevant for TFBSs determined by high-throughput techniques and made available in general repositories such as GEO (Barrett et al., 2013) and ArrayExpress (Athar et al., 2019) or in specialized portals such as ENCODE (Contrino et al., 2012). Even so, it is still difficult and expensive to prove that any TFBS is involved in the regulation of a gene. To overcome the lack of tools to assign TFs to the regulation of their target genes, we propose a two-step approach to both improve and automate the assignation of TF to the regulation of target genes. The first step of our methodology assigns TF to genes employing a distance threshold between ChIP-Seq derived TFBSs and genes, creating a GRN that over-estimates targets for each TF (Chen et al., 2020). Then, in a second step, this initial GRN is filtered by using a large collection of RNA-Seq data and GENIE3, but instead of using this tool to select regulators from all TFs in the genome for each gene, we use it to select regulators from all TFs assigned to a gene in the first step.

To demonstrate the improved consistency of resulting networks we employed *D. melanogaster* because of its relatively small genome and the availability of experimentally determined TFBS for many TFs. Based on that, TFs that regulate the same gene tend to interact between them (Shokri et al., 2019), forming the so called transcriptional complex (Ogata et al., 2003), we will show how our approach provides an effective method to increase the reliability of TF target assignments. In this way, one expects an increase on the connectance in interaction networks made of all TFs regulating the same gene after using our approach. In addition, as a case example to show the utility of our approach, we studied the role of *D. melanogaster* gene *Hr96* (UniProt Q24143) in the transcriptional control of mitochondrial genes. *Hr96* is a TF orthologous to the human Vitamin D receptor (Fisk and Thummel, 1995). *Hr96* is activated by small lipophilic compounds from dietary signals and metabolic intermediates, acting in the regulation of developmental pathways and cellular metabolism (McKenna and O'Malley, 2002). It is mainly expressed during the mid-embryogenesis stages in the metabolic fat body, excretory organs, and in the central nervous system (Wilk et al., 2013), mostly induced by the ecdysone hormone, the main factor that coordinates molting and metamorphosis (Fisk and Thummel, 1995). *Hr96* plays a role in xenobiotics detection such as the pesticide DDT and phenobarbital, inducing the expression of detoxification and clearance genes (King-Jones et al., 2006). Furthermore, *Hr96* has a key role in lipid metabolism, sensing triacylglycerol levels to facilitate their breakdown, and regulating cholesterol catabolism through modulation of genes involved in its storage, uptake, and trafficking (Horner et al., 2009; Sieber and Thummel, 2009). However, despite these features, little is still known about the role of *Hr96* on the regulation of gene expression associated with mitochondrial function to directly modulate lipid and energy metabolism.

## 2. Materials and Methods

**Figure 1:**
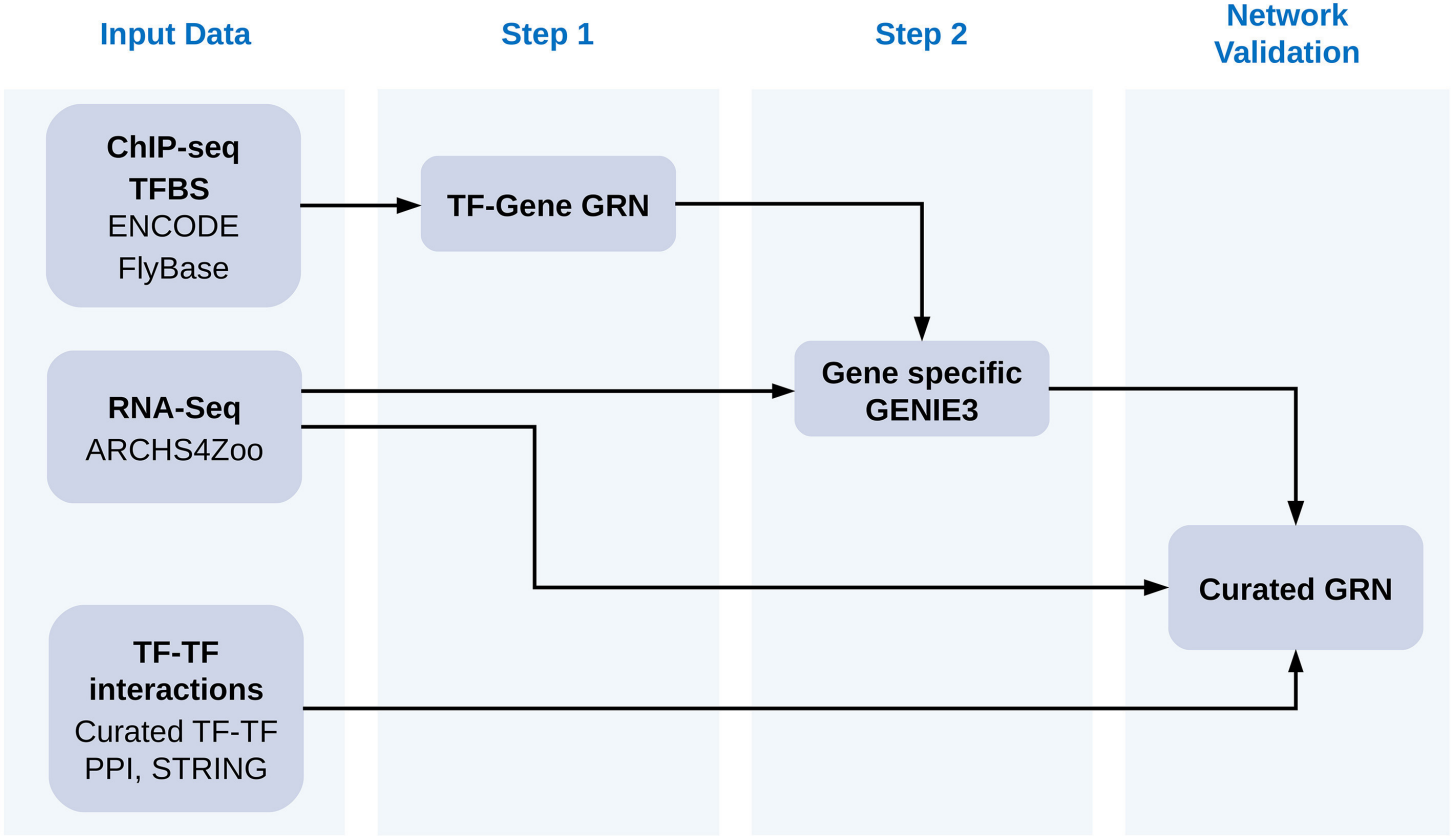
Workflow of our approach. We first gathered a collection of TFBS from ENCODE and FlyBase determined with ChIP-Seq experiments and used them to assign TF to the regulation of specific genes according to their distance to genes. We then used GENIE3 to prune TFs for each gene. We employed as input for GENIE3 all gene counts available for *Drosophila melanogaster* at the ARCHS4ZOO repository for all TFs assigned to the same gene in the first step. We then demonstrated how the results of pruning TF-gene assignments improved the resulting gene regulatory networks by increasing the connectance in the TF-TF interaction networks made of all regulators for the same gene. We employed TF-TF interactions from a curated yeast two hybrids collection, from TF-TF interactions obatined at the STRING database and from TF-TF coexpresion networks calculated from ARCHS4ZOO gene counts. Additionally we also demonstrated that genes sharing more than one TF tend to have expression patterns more correlated after the second step of our approach than by simply using distance cut-offs to assign TF to genes.

The general workflow of our approach is described graphically in Figure 1. Each of the steps described in the figure and how we obtained data is explained in detail below.

### 2.1. Reference Gene Regulatory Networks

We created reference gene regulatory networks for *D. melanogaster* by combining TFBS information from the ChIP-Seq available at the ENCODE data repository (Contrino et al., 2012) and FlyBase (Thurmond et al., 2019) as were available on July 2019 and March 2020, respectively. In this way, we inferred regulatory relationships based on the distance between the ChIP-Seq determined TFBSs for a total of 350 TFs and the Transcription Start Site (TSS) of each gene in the genome of the fruit fly version 6.32. To determine whether a TF regulates a gene, we chose distance thresholds between TFBSs and the TSS of each gene, so if the TFBS falls within this distance, we assumed it regulates the respective gene. We created three reference networks with different distance thresholds: 1,500, 2,000, and 5,000 nucleotides inspired by other approaches (Dupuy et al., 2004; Blatti et al., 2015) and described in Table 1. Further details on ChIP-Seq data employed and the procedure used are available in Murgas et al. (2021).

**Table 1:** Description of the networks analyzed in this work.

	**Threshold (kb)**	**Genes**	**Edges**	**Avg. Indegree**	**Avg. Outdegree**
Reference networks	1.5	15,576	1,094,130	44.50	3,126.09
	2	15,899	1,190,168	45.43	3,400.48
	5	16,665	1,679,173	47.61	4,797.64
Filtered networks	1.5	11,635	147,203	33.24	420.58
	2	11,968	369,346	34.19	1,055.27
	5	12,994	467,442	37.13	1,335.55

*Reference networks were created by assigning TFs to the regulation of specific genes based on a distance threshold between the TFBS and the gene. Filtered networks were created by selecting the TFs for each gene that better predict its expression levels with GENIE3. All networks described in the table contain the same 350 TFs*.

### 2.2. Gene Expression Profiles and Network Inference

To obtain a comprehensive dataset of transcriptomic data, we employed all RNA-Seq experiments of *D. melanogaster* available at ARCHS4ZOO version update 8/2018 (Lachmann et al., 2018) as was available on April 2020 at https://maayanlab.cloud/archs4/archs4zoo.html. This dataset comprises 9,924 RNA-seq samples belonging to 368 series and gene counts were used as available from the data repository without further processing as previously recommended (Aibar et al., 2017). This dataset of gene expression profiles was then employed with GENIE3 (Huynh-Thu et al., 2010) to remove TF-gene regulations from the regulators assigned to each gene in the reference networks. GENIE3 employs a random forest algorithm to select the subset of TF for each gene whose expression better predicts the expression of the gene, assigning them those TFs as regulators of that gene. In our case, we created subsets of expression data with all samples for each gene and for all TFs that were assigned as its regulators using each of the three distance thresholds, and employed GENIE3 to determine which TFs better predicted the expression of the gene, and thus, were actually regulating it. GENIE3 does not use a preset cut-off to select regulators and reports the relevance of each TF sorted by decreasing values. To remove the most unlikely regulators, we implemented a dynamic threshold by which for each gene we removed all TFs with a relevance lower than 10% of that reported for the most relevant TF.

### 2.3. Improvement of TF-Gene Assignment

We measured connectance in interaction networks made of all TFs that regulate the same gene in networks before and after using GENIE3 and counted for how many genes connectance increased. We define the connectance of a network, or connectivity density, as the fraction of connections present in a network divided by the total number of edges that could take place in the network. The connectance (ρ) lies in the range [0,1], with greater values indicating that nodes are more interconnected between them than with values closer to 0. This way, to estimate the quality of a GRN relies on the fact that TFs controlling the expression of a gene are more likely to interact between them (Shokri et al., 2019).

To validate our approach, we employed several types of TF interaction networks: a curated Protein-Protein Interaction (PPI) network (Shokri et al., 2019); a correlation network calculated with Pearson's correlation coefficient on the same expression data used with GENIE3 with edges defined with different thresholds; and STRING functional networks (Szklarczyk et al., 2019) created querying this database with all 350 TFs on September 2020 and filtering the resulting network at different confidence thresholds for combined score and several evidence types on its own. These networks are described in Table 2. Additionally, we also calculated average gene co-expression for all pairs of genes regulated by at least the same two TFs. This is based on the idea that co-regulated genes should have more similar expression patterns than those which are not regulated by the same TFs (Martyanov and Gross, 2010). We calculated average Pearson correlation on the ARCHS4ZOO RNA-Seq data between pairs of genes that share more than one TF in filtered and reference networks. We assumed normality and used a two samples *T*-test to compare if the difference between the average for genes sharing the same number of regulators before and after GENIE3 was significant.

**Table 2:** Description of TF-TF interaction networks employed to verify our approach.

**Network**	**Nodes**	**Edges**
Corr 0.25	349	30,915
Corr 0.45	340	16,584
Corr 0.65	288	6,912
Corr 0.85	137	353
Curated_PPI	271	796
STRING (combined ≥ 0.5)	260	1,065
STRING (combined ≥ 0.8)	150	241
STRING (textmining ≥ 0.4)	265	1,351
STRING (textmining ≥ 0.6)	196	502
STRING (textmining ≥ 0.8)	139	223
STRING (database_annotated ≥ 0.4)	53	69
STRING (experimentally_determined ≥ 0.5)	117	120
STRING (experimentally_determined ≥ 0.7)	65	49
STRING (experimentally_determined ≥ 0.9)	26	15

*Correlation networks were created using different thresholds of positive Pearson's correlation coefficient. Curated PPI is the subnetwork of the 350 TFs employed to create our reference GRN verified experimentally in (Shokri et al., 2019). Interaction networks obtained from STRING (Szklarczyk et al., 2019) differ on the criteria employed to define edges: STRING (combined score ≥ 0.5) is the functional interaction network retrieved querying the STRING web with the 350 TF and by default parameters, i.e., combined score ≥ 0.5. All other STRING networks were created by employing different thresholds with single evidence types and thresholds applied*.

### 2.4. Hr96 and Its Role in *D. melanogaster* Mitochondrial Function

#### 2.4.1. Selection of Mitochondrial Genes and Functional Characterization

We first assigned all *D. melanogaster* genes as mitochondrial if sub-cellular localization GO terms associated to them available at FlyBase (Thurmond et al., 2019) contained the term “mitochondria.” Following, we created GRNs formed by these mitochondrial genes and all TFs in the networks using the regulations present in the global networks.

#### 2.4.2. Network Analysis, Visualization and *Hr96* Centered Subnetworks

All network analyzes were carried out using Cytoscape (Shannon et al., 2003). This platform was also employed to create subnetworks using its graphical interface as follow. Subnetworks centered on *Hr96* were created by selecting its node in each network before and after applying our procedure, and then using Cytoscape to select all nodes connected to *Hr96* by edges arising from it, i.e., regulated by *Hr96*.

## 3. Results

We first show how our approach improves the consistency of TF-gene assignment created by assigning TFs to genes if a TFBS is near the gene. Following, we demonstrate how using the improved version of the networks leads to edges that are more likely to take place, and which, in fact, allow interpretation and analysis that are precluded in unpruned networks.

### 3.1. Characterization of Networks Before and After Applying Our Approach

Table 1 shows different properties of the networks created using three distance thresholds (1.5, 2, and 5 kb) to assign TFs to the regulation of genes. First, all networks before and after applying our approach contain edges arising from all the 350 different TFs employed in this work. We then looked at the average outdegree and indegree, respectively for TF and non-TF genes in each network. These metrics, averaged connectivity for each node type, serve as indicator of how dense the networks are. While unfiltered networks have average outdegree ranging from 3,126 in the network with the more restrictive distance threshold of 1.5 Kb–4,797 in the 5 kb threshold network, the networks after using our approach have smaller values (420 with 1.5 kb–1,335 with 5 kb), evidencing a significant reduction on the number of genes regulated by the same TFs. Regarding the number of nodes that are connected by at least one edge, there is also a decrease of about 4,000 in the number of genes in the three networks and a reduction in the average indegree.

**Figure 2:**
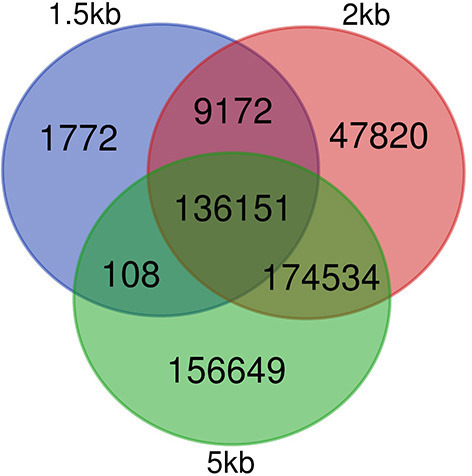
Conservation of edges in GRNs after filtering unlikely edges. Venn diagram showing edges in GENIE3 networks for each of the three distance thresholds employed, 1.5, 2, and 5 kb. Edges were defined by their source and target node IDs.

Regarding the number of TF and nodes, networks made with shorter distance thresholds are included in reference GRNs made with longer distance cut-offs before filtering. For filtered networks, this is not the case. All nodes with at least one connection in the 1.5 kb filtered network are in the network made with the 2 kb threshold, and the same occurs with nodes in the 2 and 5 kb cut-off. Nonetheless, some of the edges in the 1.5 kb network are not present in the 2 kb and the same occurs for edges in the 2 and 5 kb networks (see Figure 2). This is caused by the dependence of each edge on the expression patterns of all regulatory nodes for each gene and how GENIE3 combines them.

#### 3.1.1. Connectance Analysis on TF-TF Interaction Networks

Considering the connectance in all TF-TF subnetworks made with all regulators for each gene, there is a clear trend after applying our approach. We observe a greater number of genes with increased connectance in the TF-TF interaction network for all the regulators of each gene, see Table 3. Employing the curated PPI network, more genes show an increase in the TF connectance than genes showing a decrease in their TF connectance for all three distance cut-offs. Using the curated PPI the network with the 2 kb distance threshold has the smaller proportion of genes with decreased connectance. Using co-expression networks made at different thresholds of Pearson's correlation, the number of genes with greater connectance is notoriously larger than the number of genes with lower. As the correlation threshold used to define edges increases, the proportion of genes with smaller connectance increases as genes with greater values decrease. With STRING interaction networks and the reference network created with the 1.5 kb threshold, our approach produced TF-TF interaction subnetworks with lower values of connectance for most of the genes. In contrast, with the other two reference networks (2 and 5 kb) we also see the general trend of better connectance after our approach.

**Table 3:** TF interaction connectance comparison between networks before and after using our approach.

		**Genie3_1,500**			**Genie3_2,000**			**Genie3_5,000**			**Reference network**	
		**Better**	**Worse**	**Equal**	**Better**	**Worse**	**Equal**	**Better**	**Worse**	**Equal**	**#edges**	**#nodes**
Curated PPI		0.373	0.333	0.294	0.346	0.222	0.433	0.381	0.255	0.364	796	271
ARCHS4	corr_0.25	0.620	0.093	0.286	0.511	0.065	0.424	0.546	0.099	0.355	30,915	344
	corr_0.45	0.603	0.111	0.287	0.492	0.084	0.425	0.520	0.125	0.355	16,584	326
	corr_0.65	0.569	0.143	0.287	0.462	0.112	0.425	0.482	0.161	0.356	6,912	256
	corr_0.85	0.492	0.187	0.321	0.412	0.145	0.443	0.430	0.194	0.377	353	95
STRING	combined_0.5	0.352	0.355	0.293	0.306	0.264	0.43	0.341	0.298	0.361	1065	260
	combined_0.8	0.297	0.392	0.311	0.336	0.225	0.439	0.362	0.265	0.373	241	150
	textmining_0.4	0.344	0.366	0.29	0.285	0.287	0.428	0.317	0.323	0.36	1351	265
	textmining_0.6	0.299	0.392	0.309	0.291	0.274	0.435	0.332	0.302	0.366	502	196
	textmining_08	0.191	0.461	0.348	0.275	0.273	0.452	0.315	0.305	0.38	223	139
	experimental_05	0.184	0.468	0.348	0.3	0.241	0.459	0.333	0.278	0.388	120	117
	experimental_07	0.1	0.48	0.419	0.233	0.27	0.498	0.255	0.315	0.43	49	65
	experimental_09	0.047	0.428	0.525	0.148	0.281	0.571	0.165	0.336	0.499	15	26
	database_04	0.228	0.434	0.337	0.334	0.208	0.459	0.358	0.249	0.392	69	53

*This table shows the percentage of genes with greater connectance in the interaction network for all its TFs in all interaction networks employed to test how using GENIE3 to filter the networks improved the three GRN based on distance TF assignment (1.5, 2, and 5 kb at maximum between the TFBS and its target gene)*.

#### 3.1.2. Co-expression Analysis of co-regulated Genes

We compared the mean co-expression correlation between all pairs of genes that share at least two TFs in networks before and after filtering them with GENIE3 on the three cut-offs (See excel file provided in Supplementary Material). We found a decrease in the number of genes corregulated by the same TFs after filtering the networks, the maximum number of shared TFs between at least five pairs of genes is 25 in the filtered network at 1.5 kb while there are seven pairs of genes sharing 322 TFs before using GENIE3. Greater number of shared TFs between genes are also seen with 2 and 5 kb thresholds, but again there are less shared regulators after filtering the networks. Considering the statistical significance (*p* ≤ 0.0005) of the difference between the means, we found that in the 1.5 kb networks, pairs of genes sharing at least 2, at least 3, 4, 5, 6, 7, 8, 9, and up to 10 TFs are significantly more correlated after filtering the networks. At 2 kb cut-off, means of correlated co-expression are greater for pairs of genes sharing from 2 to 18 regulators and from 2 to 20 at 5 kb.

### 3.2. *Hr96* and Its Role in *D. melanogaster* Mitochondrial Function

**Figure 3:**
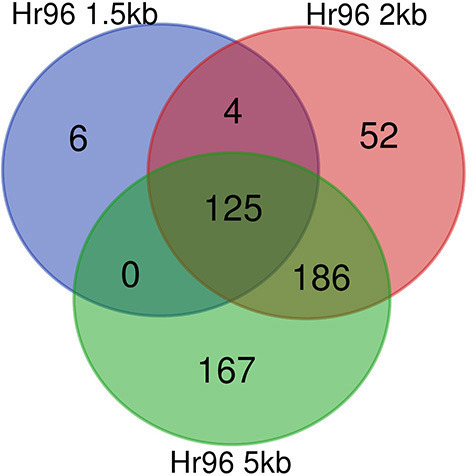
Conservation of edges in *Hr96* centered subnetworks after filtering unlikely edges. Venn diagram showing edges in GENIE3 networks for each of the three distance thresholds employed, 1.5, 2, and 5 kb. Edges were defined by their source and target node IDs.

Here we report the results of studying the subnetwork centered on *Hr96*. We first looked at the overall changes in this subnetwork before and after filtering it with GENIE3 at the three selected distance thresholds used to assign TFs to genes. We then focus on the analysis of the genes in these subnetworks. The decrease in the number of edges and nodes in the subnetworks centered on *Hr96* is evident in Table 4. This reduction in network elements is more notable regarding the number of edges, which show a reduction of more than 90% in all three networks compared to the 58–76% reduction in the number of nodes. Accordingly to what we saw on whole genome GRNs (see Table 1), there is also a large decrease in the average outdegree for TFs in the *Hr96* centered subnetworks. As to differences on the three distance thresholds, 2 and 5 kb GRNs behave more similarly between them than when compared with the 1.5 kb GRN. There are six edges exclusively in the 1.5 kb filtered subnetwork of *Hr96* which are absent in the 2 and 5 kb GRNs, and 52 nodes are present only in the 2 kb network and 167 in the 5 kb (see Figure 3). However, there is yet a trend of fewer edges in GRNs made with more stringent thresholds that in their majority appear in more relaxed cutoffs.

**Table 4:** Description of subnetworks centered on *Hr96*.

	**Before**		**After**	
**Network**	**Nodes (TFs)**	**Edges**	**Nodes (TFs)**	**Edges**
1.5 kb	191 (81)	8,840	47 (14)	135
2 kb	201 (84)	9,859	84 (17)	384
5 kb	253 (109)	17,652	98 (21)	478

*Number of nodes and edges in the subnetworks created starting from Hr96 for each of the GRNs created at different distance thresholds before and after applying our approach. The number of nodes depicting TF coding genes is between brackets*.

**Figure 4:**
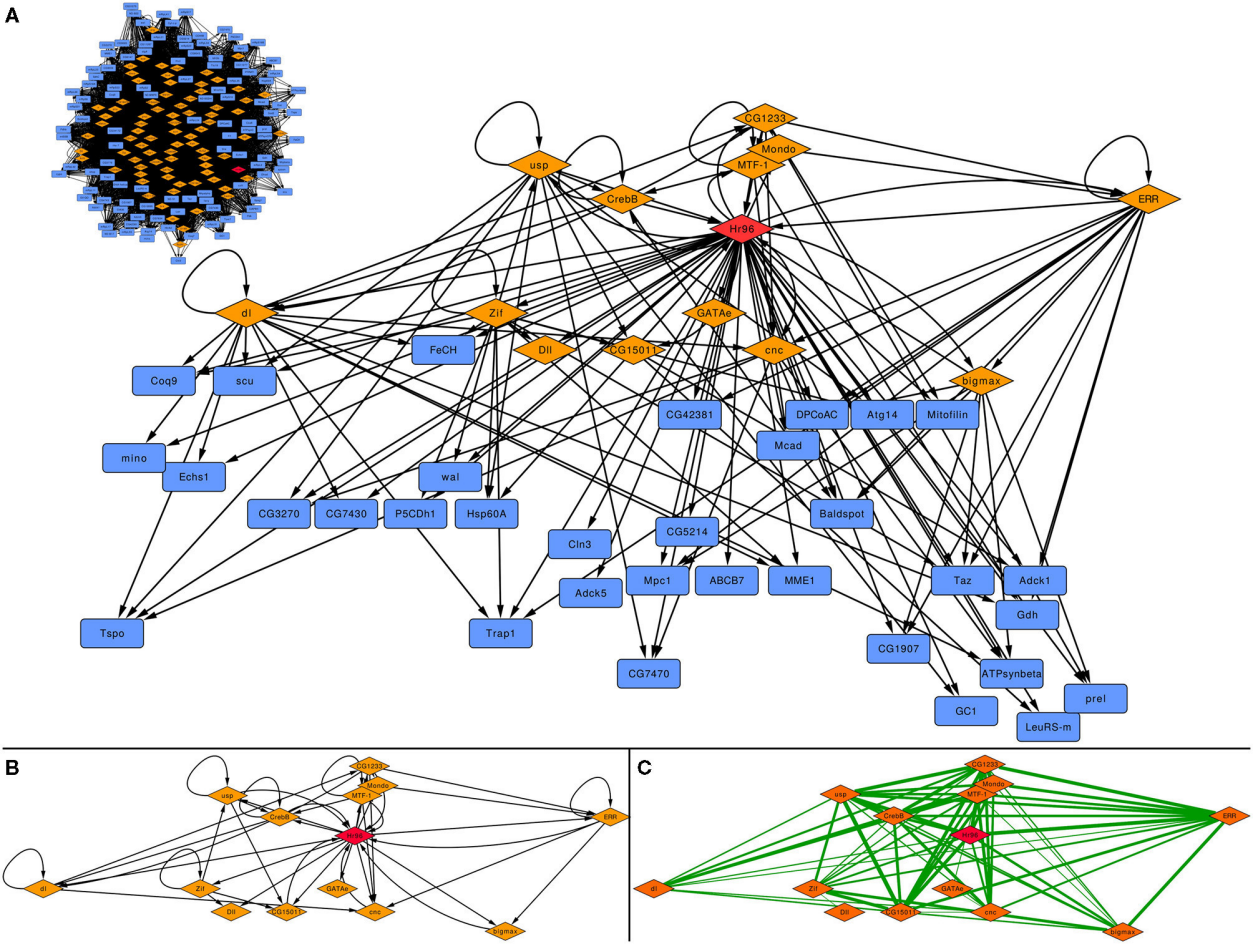
Subnetwork centred on Hr96 depicting its involvement on the regulation of mitochondrial genes. **(A)** depicts the whole subnetwork after applying GENIE3 and before (inset on the top left side of the panel); **(B)** represents the inter-regulation of the 14 TFs present in this subnetwork; and **(C)** is the TF-TF interaction network formed by the same 14 TFs where edges represent Pearson correlation calculated on the 9,924 samples for *D. melanogaster* obtained from the ARCHS4ZOO [thickness is proportional to the value of positive correlation coefficients, all in the range (0.46, 0.88)]. Orange diamonds depict TFs, blue rectangles non-TF genes and the red diamond is *Hr96*.

Based on its reduced number of nodes and edges (see Supplementary Material), we selected the subnetwork centered on *Hr96* made with the 1.5 kb threshold to study the function of this TF on the regulation of mitochondrial genes, shown in Figure 4A as well as the subnetwork generated in the same way for the 1.5 kb before applying GENIE3 as a filter (top left inset). There are only 14 TFs (all regulated by *Hr96*) that form a densely connected regulatory cascade together with 33 non-TF coding genes. Figure 4B displays how these 14 TFs are interconnected maintaining the same layout as above, while edges between these TFs in Figure 4C represent Pearson's correlation calculated using the same expression data previously employed with GENIE3, with their thickness indicating higher coefficients. There are 66 edges in the correlation network, 20 more than in the GRN made with the same TFs, indicating a strong co-expression pattern between these related TFs. The same network generated before applying GENIE3 is formed by 81 TFs and 110 non-TF coding genes (top left of Figure 4A). Using 2 kb, the network filtered with GENIE3 centered on *Hr96* contains three more TFs and 34 more non-TF genes, while before GENIE3 it has 84 TFs and 117 genes (See Supplementary Material). With the less stringent cut-off of 5 kb, the network filtered with GENIE3 is formed by 21 TFs, the 17 included in the 2 kb network plus another 4, and 77 non-TF. Before using GENIE3 on the 5 kb GRN, the subnetwork has 109 TF and 144 non-TF genes (See Supplementary material).

We then studied the function carried out by those 33 genes in the *Hr96* 1.5 kb GRN filtered with GENIE3. Among these, there are several carboxylic acid-related genes, especially involved in its transport and metabolism. This result indeed highlights the *Hr96* regulation of lipid metabolism-related targets in the mitochondria. In the glutamate and fatty acid metabolic and carboxylic catabolic processes, we found that the *Hr96*-mitochondrial network mainly links enzymes such as dehydrogenases, oxidoreductases, and a short-chain enoyl-CoA hydratase (*Echs1*).

## 4. Discussion

The control of gene transcription is one of the key processes in living organisms. Despite its relevance, we still do not know most of the specific TFs that determine which gene is expressed and which is not. Currently, high throughput techniques such as ChIP-Seq are routinely employed to annotate TFBSs, but even if this type of knowledge becomes widespread, it still remains to assign TF binding each site to the regulation of target genes. However, even if TF target assignment is carried out routinely in a low-throughput fashion for some TF-gene pairs, whole genome TF target identification remains an expensive and almost impossible task using experimental verification. Here, we propose a two step approach to address this issue: first TFs are assigned to the regulation of certain genes if ChIP-Seq derived binding sites fall within a distance cut-off to the gene. Then, in a second step, for each gene, we remove improbable regulations by using a large collection of RNA-seq data (Lachmann et al., 2018) as input for GENIE3 (Huynh-Thu et al., 2010). Instead of feeding GENIE3 with the expression of all TFs and genes, for each gene we only employed its expression and the expression of all regulators assigned to it in the first step. By doing this, we changed the purpose of GENIE3 from whole genome GRN inference to GRN pruning.

Most eukaryotic genes are regulated by more than one TF that, acting simultaneously, determine whether their target gene expresses or not. TFs, thus, interact forming transcriptional complexes (Ogata et al., 2003) in a cooperative fashion (Hancock et al., 2019) to actively control transcription. Consequently, we assumed that the actual regulatory TFs of each gene would need to interact forming an interconnected TF-TF interaction network. And thus, that the connectance of this TF-TF interaction network would increase if wrongly assigned TFs were removed from the regulation of each gene. We took advantage of a recently released, high confidence, TF-TF PPI network of *D. melanogaster* (Shokri et al., 2019) to test if the connectance between all TFs assigned to each gene increased as expected after using our approach. In addition, to demonstrate the improvement in TF-target assignment deemed to our approach, we also employed several other interaction networks obtained from STRING functional networks (Szklarczyk et al., 2019) and a co-expression network calculated with Pearson's correlation on the same transcriptional dataset employed to remove TF-gene pairs with GENIE3.

We tested if the connectance between TFs regulating the same gene increased with three different distance thresholds of 1.5, 2, and 5 kb for the initial assignation of TFs to genes (Table 3). For a 2 kb cut-off, our results indicate a consistent increase of connectance calculated for all regulators that is independent of how the interactions between TFs are defined. This tendency is almost as consistent for 5 kb and can also be seen for 1.5 kb, even if there are few exceptions for these improvement on the connectance. Importantly, these exceptions mainly appear for very stringent definitions of TF-TF interactions, such as a STRING combined score ≥0.8, or STRING experimental score ≥0.9 for all three cut-offs. Nonetheless, using the high confidence PPI network (Shokri et al., 2019) and all correlated co-expression, a majority of genes had better connectance among their regulators after using GENIE3 than without using it. Even if, biologically, it makes more sense that our approach results in higher connectance between the regulators of each gene, experimentally this can only be tested by comparing our results with a null background. In our case, this would imply the need to randomly remove TF-gene associations for each gene. Nonetheless, it is expected that as TF-TF interaction networks are very sparse, any randomly selected subnetwork is deemed to also be sparse, unless there is biological significance embebed in the approach followed to remove edges.

The observed TF-connectance improvement is more consistent if the TF interaction network has interactions for all regulators. As shown in Table 2, the network whose edges are Pearson's correlation ≥ 0.25 (*cor*_0.25) contains interactions for 349 out of 350 TFs for which there were ChIP-Seq data available and 62% of the genes show better connectance at 1.5 kb (9.3% worse), 51.1% are better at 2 kb (6.5% worse), and 54.6% at 5 kb (9.9% worse). On the other hand, using Pearson's ≥ 0.85 (*cor*_0.85) there are only interactions for 137 TFs and 49.2% of the genes showed improved TF connectance at 1.5kb (18.7% worse), 41.2% are better at 2 kb (14.5%), and 43% at 5 kb (19.4% worse). This previous example indicates that having TF interaction networks with high confidence interactions for all regulators is a key factor to consider when estimating the certainty of the improvement in connectance. It is also very important to take into consideration that a correlation ≥ 0.25 is very likely to be significant taking into account it was calculated with 9,924 expression experiments. It should also be considered that the yeast two hybrid experiment, used to determine the PPI curated network, simply does not work for some proteins or it may produce too many false positive or false negative hits (Koegl and Uetz, 2007), and thus, careful curation is indispensable. Similarly, STRING networks are automatically generated and their scores are calculated without any human intervention, making it desirable to carry out manual inspection of each edge and its supporting evidence before using it. Importantly, the results we obtained from the analysis of co-expression between gene pairs that share the same number of TF before and after filtering the networks, also support that our approach does indeed improve the reliability of TF-gene assignment (see excel file in the Supplementary Material). These results also showed a notable decrease in pairs of genes that share large numbers of regulators (more than 25 shared TFs), which is caused by the reduction on the number of TF-gene assignments.

We then focused on the subnetwork centered on a specific TF to showcase the utility of the networks generated by our approach. Nuclear hormone receptors (NHR) represent a key hub in the regulation of development, reproduction, and metabolism (Fahrbach et al., 2012). Most NHRs are ligand-regulated TFs activated by lipophilic ligands such as steroid hormones, fatty acids, phospholipids, bile acids, vitamins, and xenobiotics (Huang et al., 2009). Humans present 48 NHR that, despite being widely explored in terms of structure and function, are not fully characterized (Evans and Mangelsdorf, 2014). Approximately half of those remain orphan receptors, a fact that imposes great difficulty to crack down their regulatory network (Weikum et al., 2018). In contrast, the *D. melanogaster* genome carries only 18 nuclear-receptor genes, which represent all six NHR mammalian subfamilies, but importantly showing lower functional redundancy (King-Jones and Thummel, 2005; Palanker et al., 2006). Among *Drosophila* NHRs, *Hr96* (UniProt Q24143) is an interesting case due to its orthology with three vertebrate NHR: Vitamin D Receptor (VDR) (Fisk and Thummel, 1995), Pregnane X Receptor (PXR), and Constitutive Androstane Receptor (CAR) (Hoffmann and Partridge, 2015).

VDR (UniProt P11473) is widely distributed in mammal tissues (Eyles et al., 2005) and exerts transcriptional control, influenced by vitamin D, in over 3% of the human genome (Ramagopalan et al., 2010; Shirvani et al., 2019). The control that VDR exerts on gene regulation is significantly enriched over the immune functions, cell cycle activity, DNA replication, stress response (Hossein-nezhad et al., 2013) and, also significantly contributes to mitochondrial transcriptional regulating, biogenesis, and metabolism (Lee et al., 2008). Specifically, human skeletal muscle cells treated with the VDR-ligand 1α,25(OH)2D3 showed increased mitochondrial oxygen consumption rate and network mass by down-regulating fission proteins Drp1 and Fis1, and up-regulating the fusion protein OPA1 and the mitochondrial biogenesis modulators MYC, mitogen-activated protein kinase 13 (MAPK13), and endothelial PAS domain-containing protein 1 (EPAS1) (Ryan et al., 2016). In contrast, VDR silencing appears to cause a reduction in cellular respiration, ATP production (Ashcroft et al., 2020) and induces ROS production by up-regulating cytochrome C oxidase subunits proteins (COX2; COX4) and ATP synthase subunits (ATP5B; ATP6), which enhance respiratory membrane potential leading to protons leakage (Ricca et al., 2018). In this way, to test the hypothesis that *Hr96* has the potential to regulate mitochondrial function and improves lipid-based energy production, we used our hybrid protocol to showcase its ability to improve TF factor target assignments.

We analyzed all 33 *Hr96* targeted genes that do not code for TF in the curated 1.5 kb *Hr96* network to further characterize the role of this TF in any specific process. It is important to highlight here that 32 of these genes where also present in the 2 and 5 kb curated subnetworks. In addition, we also disregarded other genes also regulated by the other 13 TFs that are also present in the subnetwork, trying in this way to emphasize the role of this NHR.

The Delta-1-Pyrroline-5-carboxylate dehydrogenase 1 (*P5CDh1*) and Glutamate dehydrogenase (*Gdh*) are enzymes that support energy metabolism by glutamate and α-Ketoglutarate production, to promote the mitochondrial respiration (He and DiMario, 2011; Hohnholt et al., 2018). As well Adck1, which is essential to keep mitochondrial structural organization, energy, and ROS production under control (Yoon et al., 2019). β-oxidation, the catabolic pathway that breaks down fatty acids in the mitochondria, is highly represented in the *Hr96*-network by different genes. Scully (*scu*) and *Mcad* catalyze two different β-oxidation enzymatic steps and are highly conserved (Torroja et al., 1998; Lim et al., 2018). The *wal* gene encodes an electron transfer flavoprotein subunit that works as a specific electron acceptor in the mitochondrial fatty acid β-oxidation of fatty acids (Alves et al., 2012; Chokchaiwong et al., 2019), while ECHS1 is shown to be involved in the second step of mitochondrial β-oxidation (Hirai et al., 2001; Al Mutairi et al., 2017). All these targets operate to maintain the respiratory chain and energy production through carboxylic acid metabolism. To our knowledge, the activity of these enzymes has not been related to *Hr96* until now. In the same line, *Hr96* modulates the Minotaur (*mino*) activity, a conserved glycerol-3-phosphate O-acyltransferase responsible for triglycerides synthesis and lipid droplets biogenesis (Fantin et al., 2019). It has been shown that when this enzyme is down-regulated as observed upon bacterial infection, there is a progressive loss of lipid energy stores (Dionne et al., 2006), meanwhile, its expression is increased in the face of starvation (Fujikawa et al., 2009) possibly promoting a mitochondrial adaptation toward lipid metabolism.

*Baldspot* (Elovl6) is another fatty acid-related gene regulated by *Hr96*. The Elov16 enzyme extends C16 fatty acids to C18. It has been shown that flies lacking Elovl6 present impaired mitochondrial respiration by promoting a hyper-fragmentation of the mitochondrial network through JNK signaling and mitofusin ubiquitination (Senyilmaz et al., 2015). Regarding anion transport, to properly regulate the mitochondrial β-oxidation, *Hr96* seems to also coordinate the transcription of carboxylic acid transport targets such glutamate carrier (GC1), mitochondrial pyruvate carrier (*mpc1*), and *Cln3*, the Batten disease-associated gene involved in arginine transport and mitochondrial β-oxidation support (Dawson et al., 1996; Chan et al., 2009). Among those, MPC1 has an important role in mitochondrial function since it is found in the inner mitochondrial membrane, and mutant *D. melanogaster* for *mpc1* display impaired pyruvate metabolism, leading to a shortage of intermediates necessary for the tricarboxylic acid cycle, ultimately reducing ATP production (Bricker et al., 2012; Tang, 2019; Rossi et al., 2020). These findings are in line with the most recent research on *Hr96* functionality that points toward its relevance in the regulation of sterol trafficking, housing, and consumption (Sieber and Thummel, 2012). Considering our analyzes, it is possible to postulate that *Hr96* also regulates triacylglycerol metabolism by modulating the transcription of mitochondrial genes to stimulate lipid consumption and mitochondrial respiration to increase ATP production.

Altogether, this analysis highlights the potential effect of Hr96 on key mitochondrial processes such as the catabolism and transport of fatty acids and small molecules.

## 5. Conclusion

We created a two-step approach with the main purpose of helping to assign TF to the regulation of specific genes. We demonstrated that the consistency of TF-gene assignment improves by increasing the number of TFs targeting the same gene that are known to interact between them. In the process of testing our approach, we investigated several distance thresholds to assign TFs to genes. Based on how the number of edges in a GRN varies more by increasing the cut-off distance between the TSS of each gene and the TFBS from 1.5 to 2 kb than by increasing it from 2 to 5 kb, we can say that the best cut-off tested was 2 kb, better than to 1.5 or 5 kb. Our results also indicate that the TF-TF interaction networks are incomplete, and that even if our current results indicate in improvement in TF-gene assignment, more complete interaction networks would help in producing more reliable GRN.

Regarding the example case of *Hr96*, our analysis provides a rational framework for further investigations on *Hr96*-mitochondrial transcriptional regulation and offers an opportunity to explore a better understanding of *Drosophila* lipid metabolism and signaling pathways for disease mechanisms.

As a final remark, our work proves that the integration of data from different sources is key to produce high quality GRNs, and thus, public data availability must be mandatory for all experimental results.

## Data Availability Statement

Publicly available datasets were analyzed in this study. This data can be found here: *D. melanogaster* Gene counts employed were downloaded from https://maayanlab.cloud/archs4/archs4zoo.html. All GRNs and a Cytoscape session with mitochondrial networks can be found here https://figshare.com/projects/Filtering_of_datadriven_gene_regulatory_networks_using_Drosophila_melanogaster_as_a_case_study/95885. All code employed in this work is now available at https://github.com/networkbiolab/Network-Filtering.git together with a README file explaining all details.

## Author Contributions

YC-A and GG carried out most of the analysis performed. LM created the GR networks. CS and MS participated in the selection and analysis of *Hr96*. AM had the initial idea, designed the filtering approach, performed *in-silico* experiments and coordinated all people involved in the project. All authors wrote the manuscript.

## Conflict of Interest

The authors declare that the research was conducted in the absence of any commercial or financial relationships that could be construed as a potential conflict of interest.

## Publisher's Note

All claims expressed in this article are solely those of the authors and do not necessarily represent those of their affiliated organizations, or those of the publisher, the editors and the reviewers. Any product that may be evaluated in this article, or claim that may be made by its manufacturer, is not guaranteed or endorsed by the publisher.
