# Supplementary material for: Filtering of Data-Driven Gene Regulatory Networks Using Drosophila melanogaster as a Case Study
Source: Front Genet. 2021 Jul 28;12:649764. doi: 10.3389/fgene.2021.649764 (PMC8355599; doi:10.3389/fgene.2021.649764)
Supplement: Supplementary file 2 [file Data_Sheet_1.pdf]

Supplementary Material

1 CONNECTANCE COMPARISONS

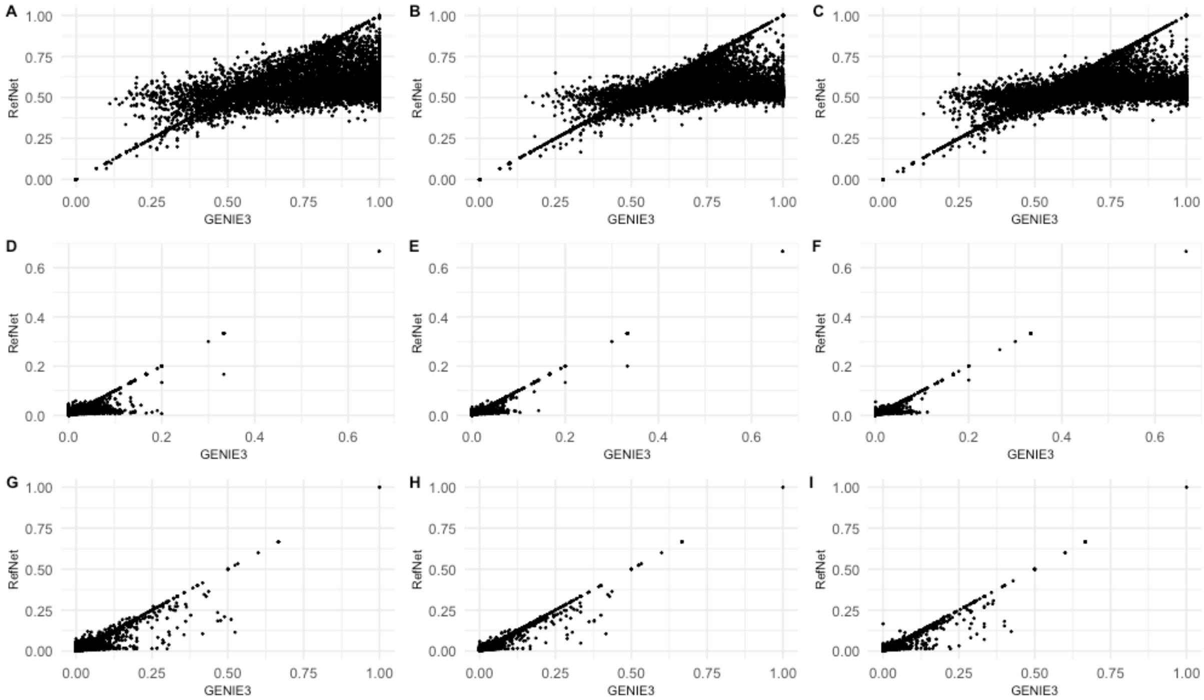

Figure S1. Comparisons of connectance for all regulatory TFs for each gene before and after curation with GENIE3. First row, panels **(A)**, **(B)** and **(C)** compare the connectance before (Y axis) versus after (X axis) respectively at 1.5, 2 and 5kb distance cut-offs using the correlation network with Pearson's ≥ 0.25 to define interactions between TFs. Second row, panels **(D)**, **(E)** and **(F)** are respectively the comparison of connectance before (Y axis) versus after (X axis) at 1.5, 2 and 5kb distance cut-offs using the curated yeast two hybrids to define interactions between TFs. Third row, panels **(G)**, **(H)** and **(I)** are respectively the comparison of connectance before (Y axis) versus after (X axis) at 1.5, 2 and 5kb distance cut-offs using the STRING combined score ≥ 0.5 to define interactions between TFs. These three networks are described in the main text. All 9 panels show a consistent increase in connectance after the curation performed with our approach (more dots in bottom right half of the plot). Dots in the diagonal represent genes with no change in connectance and those on the top left genes with greater connectance before curation with GENIE3.

2 MITOCHONDRIAL NETWORKS

	Threshold (kb)	Genes	Edges	Avg. Indegree	Avg. Outdegree
Reference networks	1.5	783	73074	93.33.5	208.78
	2	788	77533	98.39	221.53
	5	799	102356	128.11	292.45
Filtered Networks	1.5	771	9847	12.77	28.13
	2	777	24062	30.97	68.75
	5	790	24814	31.41	70.90

Table S1. Description of the networks describing how genes related to mitochondria are regulated before and after using our approach. All networks described in the table contain the same 350 TFs.

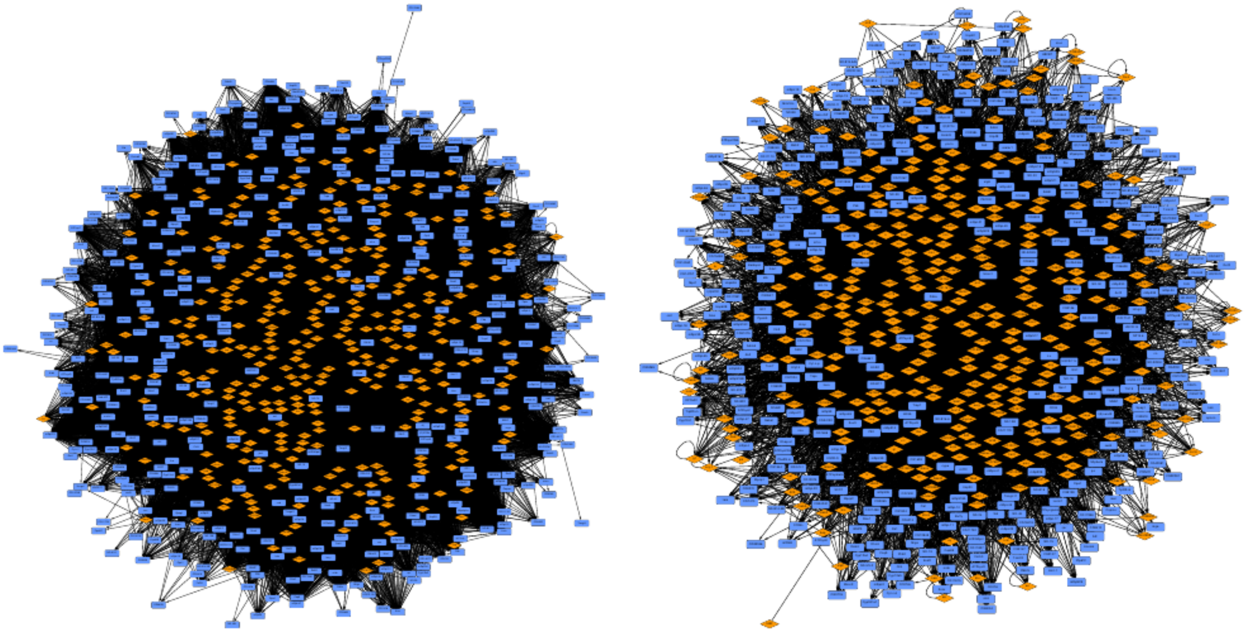

Figure S2. Mitochondrial GRN made at 1.5kb cutoff before applying the GENIE3 filter (left) and after (right). TFs are orange diamond, non-TF genes are blue rectangles.

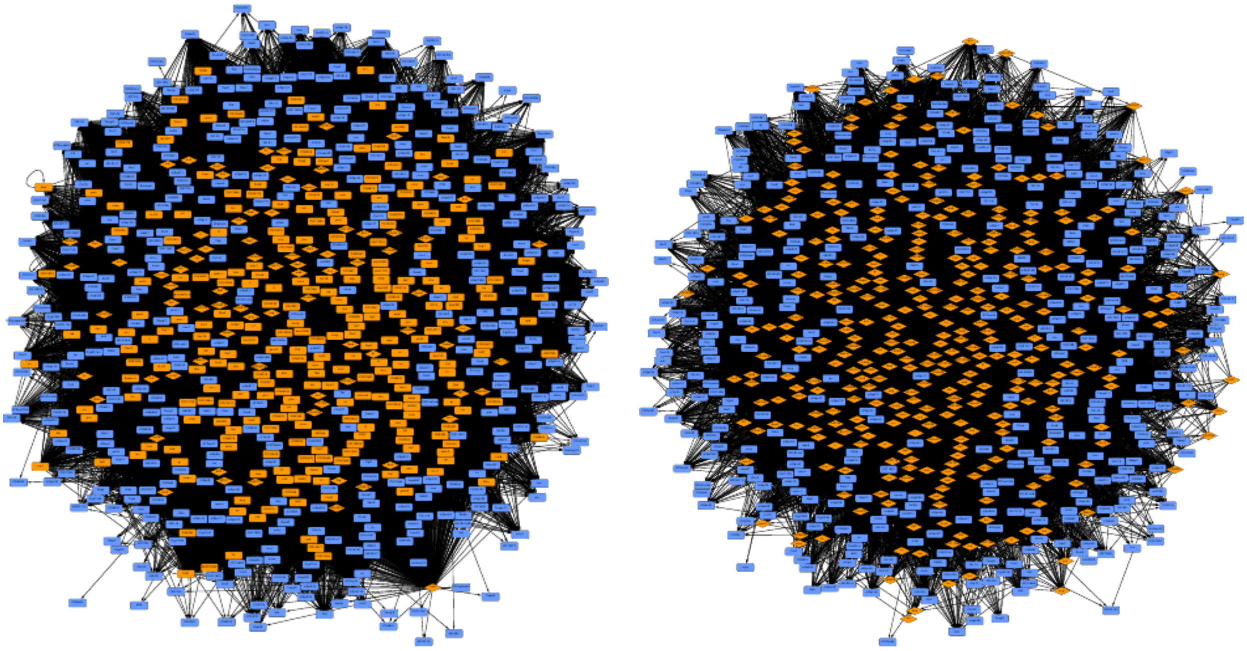

Figure S3. Mitochondrial GRN made at 2kb cutoff before applying the GENIE3 filter (left) and after (right). TFs are orange diamond, non-TF genes are blue rectangles.

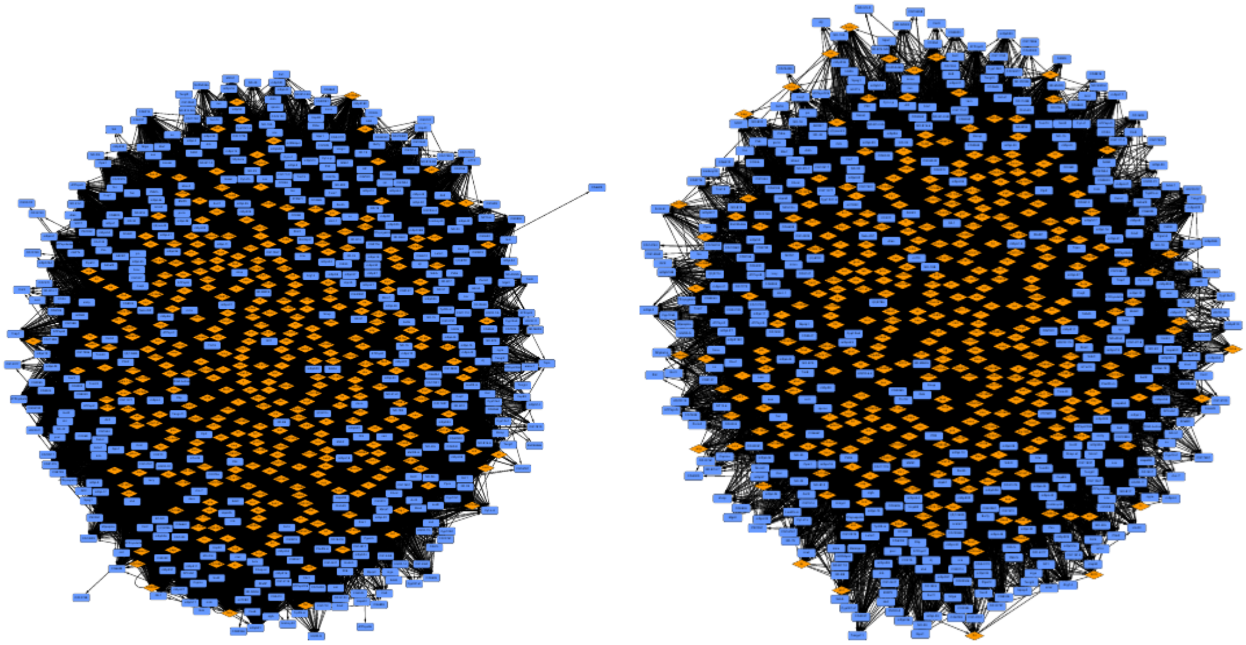

Figure S4. Mitochondrial GRN made at 5kb cutoff before applying the GENIE3 filter (left) and after (right). TFs are orange diamond, non-TF genes are blue rectangles.

3 HR96 CENTERED SUBNETWORKS OF MITOCHONDRIAL GENES

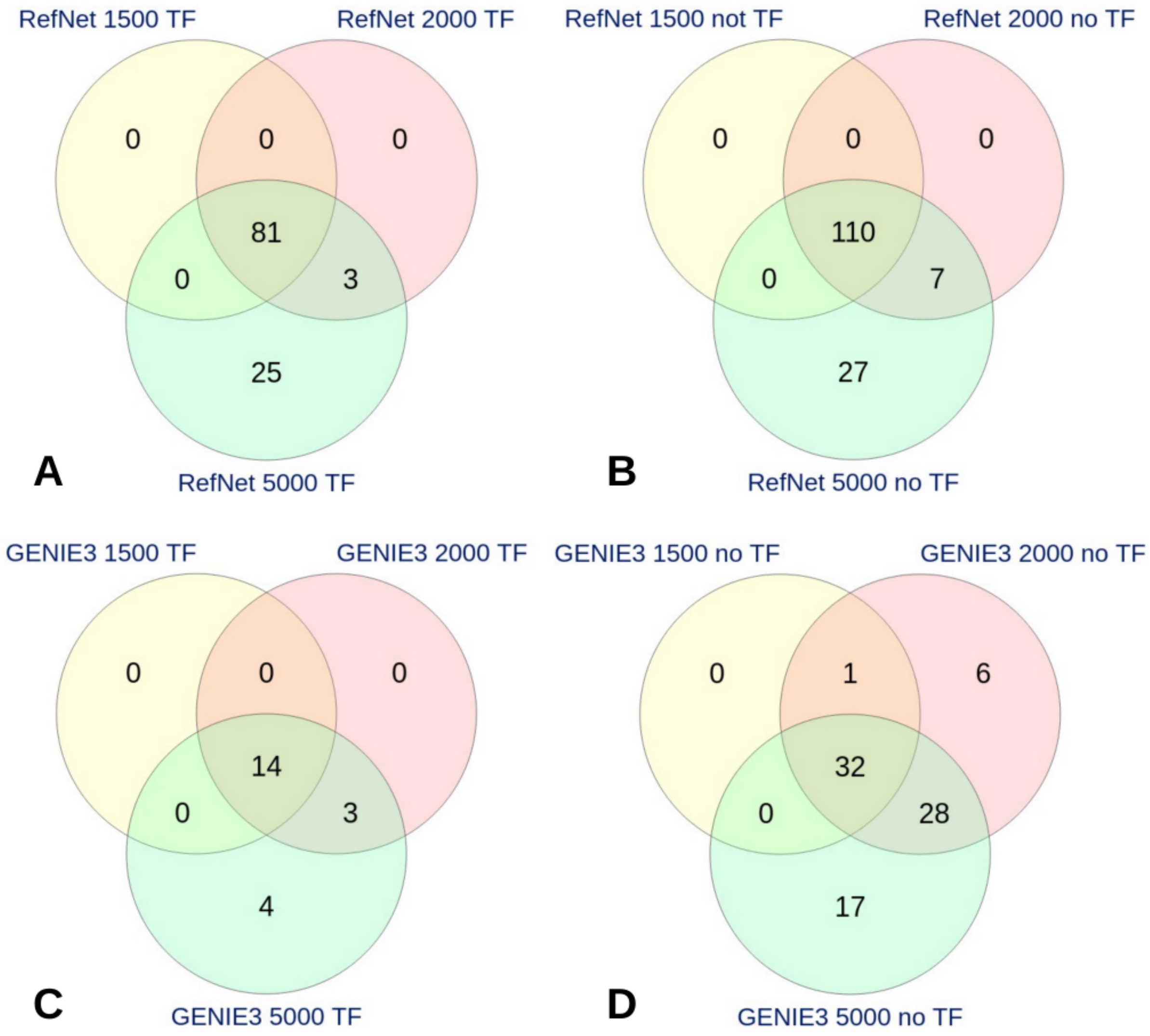

Figure S5. Comparison of the number of TFs and non-TF genes shared between filtered (GENIE3) and unfiltered subnetworks (RefNet) centred on Hr96. Number of TFs (panel A) and non-TF coding genes (panel B) shared between the three considered reference networks; and Number of TFs (panel C) and non-TF coding genes (panel D) shared between the three filtered networks Repeat as necessary for each of your figures

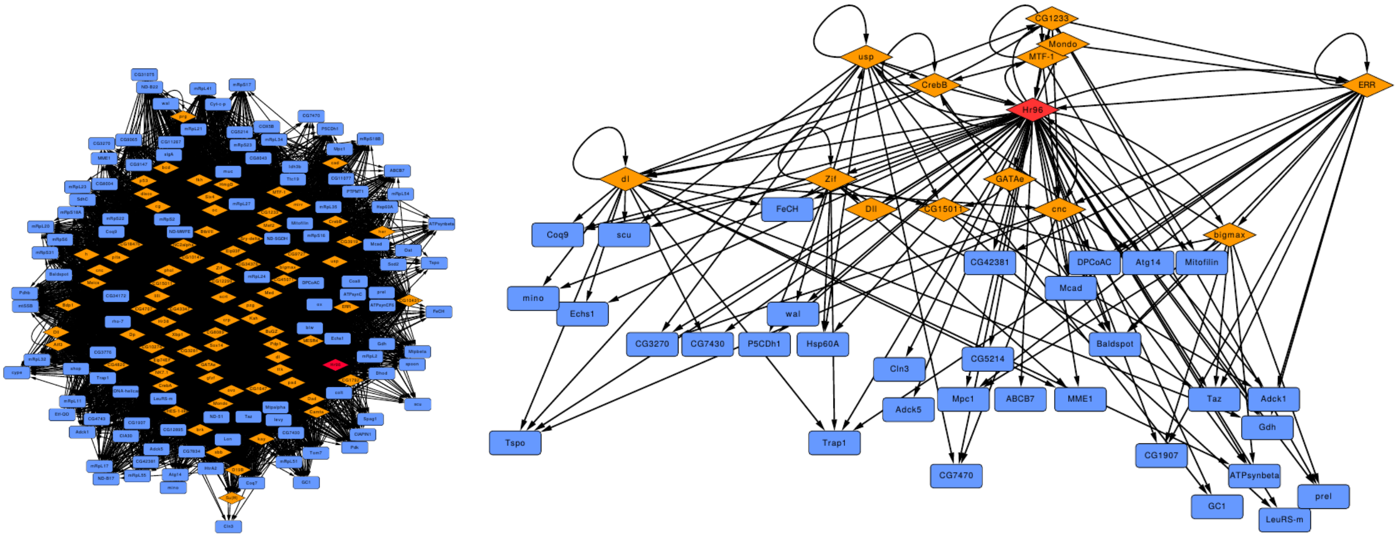

Figure S6. Mitochondrial GRN centred on Hr96 made at 1.5kb cutoff before applying the GENIE3 filter (left) and after (right). TFs are orange diamond, non-TF genes are blue rectangles. Hr96 is marked in red

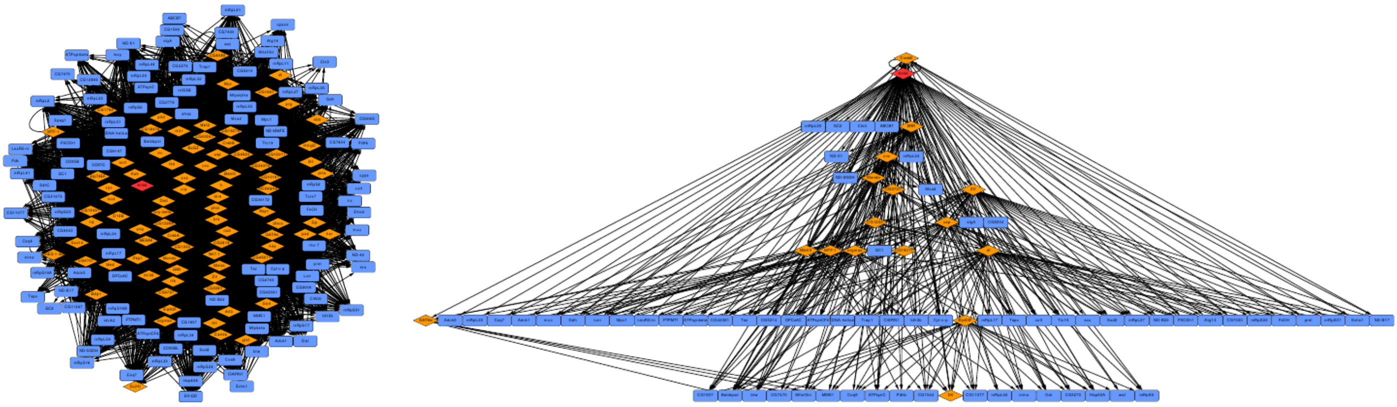

Figure S7. Mitochondrial GRN centred on Hr96 made at 2kb cutoff before applying the GENIE3 filter (left) and after (right). TFs are orange diamond, non-TF genes are blue rectangles. Hr96 is marked in red

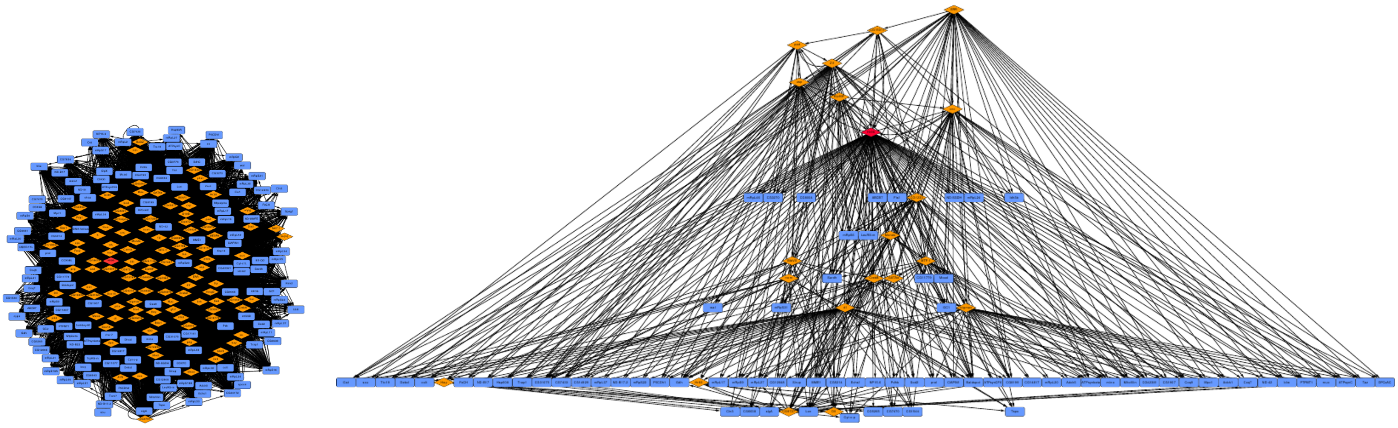

Figure S8. Mitochondrial GRN centred on Hr96 made at 5kb cutoff before applying the GENIE3 filter (left) and after (right). TFs are orange diamond, non-TF genes are blue rectangles. Hr96 is marked in red
